# Towards clinically actionable digital phenotyping targets in schizophrenia

**DOI:** 10.1038/s41537-020-0100-1

**Published:** 2020-05-05

**Authors:** Philip Henson, Ian Barnett, Matcheri Keshavan, John Torous

**Affiliations:** 1 Department of Psychiatry, Beth Israel Deaconess Medical Center, Harvard Medical School, Boston, MA USA; 2 Division of Biostatistics, Department of Biostatistics, Epidemiology, and Informatics, University of Pennsylvania Perelman School of Medicine, Philadelphia, PA USA

**Keywords:** Biomarkers, Schizophrenia

## Abstract

Digital phenotyping has potential to quantify the lived experience of mental illness and generate real-time, actionable results related to recovery, such as the case of social rhythms in individuals with bipolar disorder. However, passive data features for social rhythm clinical targets in individuals with schizophrenia have yet to be studied. In this paper, we explore the relationship between active and passive data by focusing on temporal stability and variance at an individual level as well as large-scale associations on a population level to gain clinically actionable information regarding social rhythms. From individual data clustering, we found a 19% cluster overlap between specific active and passive data features for participants with schizophrenia. In the same clinical population, two passive data features in particular associated with social rhythms, “Circadian Routine” and “Weekend Day Routine,” and were negatively associated with symptoms of anxiety, depression, psychosis, and poor sleep (Spearman ρ ranged from −0.23 to −0.30, *p* < 0.001). Conversely, in healthy controls, more stable social rhythms were positively correlated with symptomatology (Spearman ρ ranged from 0.20 to 0.44, *p* < 0.05). Our results suggest that digital phenotyping in schizophrenia may offer clinically relevant information for understanding how daily routines affect symptomatology. Specifically, negative correlations between smartphone reported anxiety, depression, psychosis, and poor sleep in individuals with schizophrenia, but not in healthy controls, offer an actionable clinical target and area for further investigation.

## Introduction

Schizophrenia affects nearly 20 million people worldwide and presents substantial health, social, and economic burdens due to the presence of comorbidities, long duration of illness, ever-present risk of relapse, and excess early mortality^1–3^. Recently, the World Health Organization created the Mental Health Gap Action Program to address this global burden of disease and provide better access to care to those in need^4^. Technology has shown promise in its utility toward healthcare access, but in order to meet the growing need, there is an urgent need for new treatments that do not merely offer a digital translation of existing therapies but instead provide novel synergies^5^.

The potential of technology to drive mental health innovation is fueled by the increased prevalence of smartphones and recent advances in mobile technology. Sensors on phones are now capable of collecting, storing, and processing vast amounts of health data, with new tools constantly emerging to help track, monitor, and augment clinical interventions. These innovations bring with them the opportunity for real-time assessment of behavior and cognition, which are important among individuals with schizophrenia where the risk of relapse is ever-present yet remains challenging to identify^6^. Using such data to inform just-in-time adaptive interventions for mental health^7^ may increase access to evidence-based care, although to date, research and products have focused primarily on mood disorders^8^. Fortunately, the same principles of real-time active monitoring of symptoms conducted through ecological momentary assessment and passive monitoring via automatically collected phone data (e.g. daily distance traveled) may offer potential for similar adaptive interventions for psychotic spectrum illnesses like schizophrenia.

Passive data collection, also known as digital phenotyping, holds clear potential to capture the lived experience of patients with mental health conditions. For example sleep disturbance is a warning sign for relapse in schizophrenia and can be captured through passive data signals such as smartphone accelerometers to capture phone movement and wearable sensors to inform duration and quality of sleep^9^ The potential of passive data to understand functional outcomes in schizophrenia is reflected in numerous ongoing studies collecting a range of sensor data from participants^10,11^ and others identifying specific correlations between phone use patterns, persecutory delusions^12^, and relapse^13^. Using these data to inform care and improve outcomes requires first that its validity be established and second that clinically actionable insights be offered to both clinicians and patients.

**Fig. 1:**
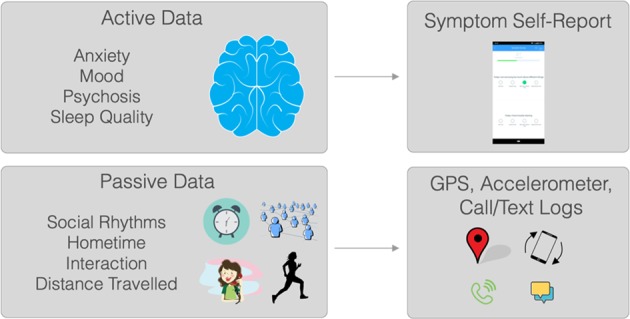
Smartphone data. Active and passive data streams measured by smartphones.

In bipolar disorder, social rhythm therapy^14^ offers a useful example of how smartphone active and passive data can be utilized to improve outcomes. The social zeitgeber model of bipolar disorder proposes that “changes in daily social rhythms or schedules lead to disruption of circadian rhythms and, in turn, onset of bipolar mood episodes”^15,16^ with research supporting that “low social rhythm regularity is a vulnerability factor for bipolar spectrum disorders”^16^. Data from smartphones (Fig. 1) can help inform social rhythms in patients with mental illness, as seen from ongoing research^17^, and commercial efforts^18^, although to date the validity of these findings for schizophrenia has not been explored. While social rhythm therapy is not itself designed for schizophrenia, understanding the feasibility of capturing these rhythms and comparing results to research in bipolar and depressive disorders offers a first step in exploring how new smartphone data streams may inform a new generation of therapies.

The ability to gather both passive and active data from smartphones often belies the complexity of these data and issues surrounding its validity. Thus, any attempt to make smartphone data clinically actionable must assess results compared to prior work and offer others the ability to replicate results. A recent systematic review on studies that collected sensor data and depression symptoms identified 85 different features (e.g. amount of home stay, sleep duration, phone calls received) across 46 studies^19^. While not all features showed consistent significance across studies, some features like sleep duration and distance traveled showed strong but opposite associations when comparing clinical and non-clinical populations. To date, there are no studies that investigate social rhythm disruption via smartphone in individuals with schizophrenia. In our prior research we have shown it is feasible to collect both active and passive data in schizophrenia including those metrics outlined above as well as measures of social rhythm via circadian routine and weekend routine derived from geolocation data^20^.

Understanding how passive data relates to active data and patient reported outcomes is a first step in assessing the utility of these data. A recent review of passive data studies across neuropsychiatric illness also noted that it is not clear if passive data are directly picking up symptomatology separate from behavioral or lifestyle changes^11^. While several studies have explored the correlations between active and passive data in sleep in schizophrenia^21,22^, results have yet to be replicated. A further challenge is that many studies with passive data have not featured a control group which makes it challenging to determine if any resulting digital signal is specific to a mental health condition or rather a broader signal associated with daily life.

To explore this relationship between active and passive data in schizophrenia and understand its potential towards social rhythm therapy, we focus on temporal stability and variance at an individual level as well as large-scale associations on a population level. We hypothesize that, as in bipolar disorder, increased stability in social rhythm (via circadian routine and weekend routine) will be associated with improved symptom scores in schizophrenia and that trends in smartphone passive data related to sociability, mobility, and physical activity in patients versus controls will align with prior research.

## Results

### Clustering

**Fig. 2:**
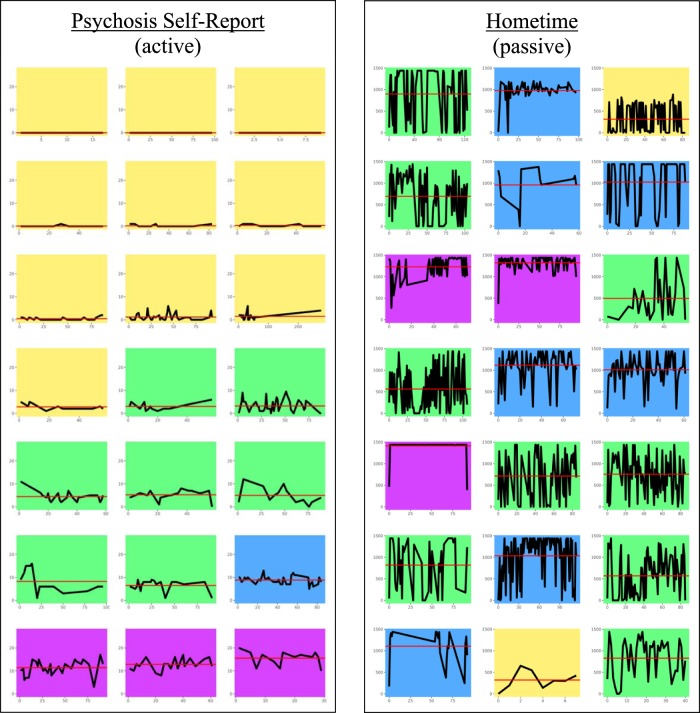
Cluster analysis. This figure compares the mean/variance of an active data stream (psychosis self-report, left) with the mean/variance of a passive data stream (Hometime, right). Each miniature plot represents a participant with schizophrenia and participants are in the same order for each set of plots. The *x*-axis represents days enrolled in the study and the *y*-axis represents magnitude of either survey response score (left) or minutes at home (right). Colors represent clusters of increasing mean/variance from yellow to green to blue to purple. Note that few participants wound up in the same cluster for their passive data as they did for their active data.

We modeled the graphical output of our k-means clustering results on a similar analysis on suicidal thoughts reported via an app^23^ using various colors to denote specific clinical clusters. Figure 2 shows an example comparing active data mean and variance (psychosis score) with passive data mean and variance (Hometime) within subjects. Only SZ participants with more than 5 days’ worth of combined passive and active data were chosen for analysis. The clusters were distinguished by color and increasing mean/variance, from yellow (lowest mean/variance) to green to blue to purple (highest mean/variance). 19% of participants (4 out of 21) were in the same cluster for active and passive and there was no overlap among participants in the cluster with the greatest mean/variance (purple).

### Correlation matrix

**Fig. 3:**
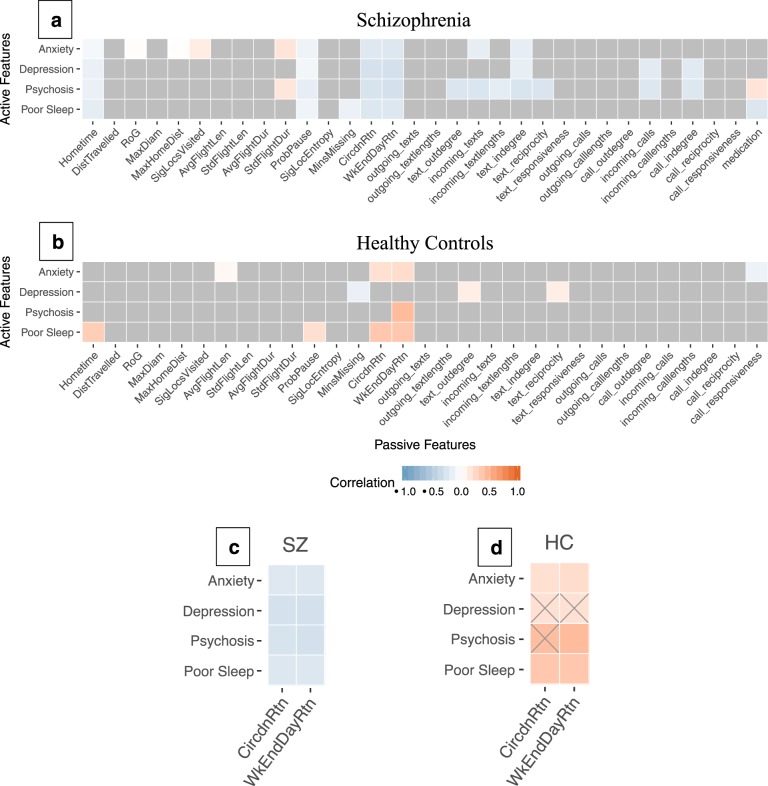
Correlation Heatmap. Correlation heatmap of passive (*x*-axis) and active (*y*-axis) features averaged from daily calculations for each participant (**a**, **b**). Gray squares in (**a**) and (**b**) indicate non-significant correlations and are reidentified as gray X’s in (**c**) and (**d**) to show underlying correlation trend direction. Passive features are discrete measures of mobility and sociability processed from raw GPS data and call/text logs. Detailed descriptions for each feature have been published by Barnett et al^24^. A focus on social rhythm features is magnified in (**c**) and (**d**).

For a broader perspective of the interaction between active and passive data, a correlation matrix was created for all the data features^24^ with the goal of identifying correlation patterns both within and between SZ and HC groups. Spearman correlation coefficients were calculated as were FDR-adjusted p-values. Figure 3 shows a portion of the matrix directly comparing active and passive data features for all 88 participants. Only correlations with an adjusted *p*-value of < 0.05 (FDR corrected) are shown. Multiple significant correlations were found in both groups with many more significant associations among features in SZ than HC. Also, significant correlations that were shared between the groups often displayed reversed polarity. A focus on the social rhythm features “Circadian Routine” and “Weekend Day Routine” in **3c** and **3d** shows an area of statistically significant correlation for both SZ and HC with Spearman ρ values ranging from −0.23 to −0.30 in SZ (*p* < 0.001) and from 0.20 to 0.44 in HC (*p* < 0.05).

As a secondary analysis, we performed hierarchical clustering on all passive data features to visualize clusters and compare those between SZ and HC. Results are shown in Supplementary Methods.

## Discussion

Smartphone digital phenotyping is able to detect changes in social rhythms that separate those with schizophrenia from controls. Greater dysregulation in social rhythms, suggesting less routine, correlated with higher severity of self-reported depression, anxiety, psychosis, and sleep symptoms in schizophrenia but not controls. These results suggest clinical utility of passive data in schizophrenia with actionable to set therapeutic goals and monitor recovery as well as relapse.

Our results suggest how digital phenotyping data in mental health must be guided by theory. We found that correlations between active and passive data were heterogeneous among individuals with schizophrenia (19% agreement), suggesting that passive data are not always a direct proxy for mental health symptoms. Understanding the mechanism of action and theories relating patient behaviors to symptoms offers a roadmap to approaching these complex data as demonstrated by our results around circadian routine informed by social rhythm therapy in bipolar disorder.

Potential clinical applications of our results include population level screening to identify psychosis risk based on early detection of passive data irregularities, such as circadian routine disturbances, as well as individual level monitoring around improving social rhythms towards reducing symptom severity. While the ability to detect correlations between social rhythms and patient reported outcomes does not imply social rhythm therapy will itself be effective, it does offer the potential of applying both new and existing therapies in an outcome-driven manner where objective data can be used to understand efficacy. Already interventions like supported employment and family education/support that inherently stabilize social rhythms have proven effective in all phases of schizophrenia, meaning that digital phenotyping could at least offer a new tool to quantify patient’s response and help titrate these multifaceted psychosocial interventions.

The correlations between passive and active data identified in our study also offer a potentially useful tool to monitor healthy populations at risk for schizophrenia, as any flipping in correlations could represent a unique digital biomarker during the high risk/prodromal phase. Our results also suggest that certain digital phenotypes may emerge on the population level via clustering analysis, based on the unique patterns of clustering in SZ as compared to HC. While the clinical utility of these clusters is less clear today, they may represent digital biotypes that are useful markers to stratify current definitions of schizophrenia, much in the same way the Bipolar—Schizophrenia Network on Intermediate Phenotypes has defined three biotypes based of EEG, neuroimaging, genetic, and clinical profiles^25^.

Our results are in line with prior research as negative correlations between circadian movement (i.e. stability in circadian rhythm) and depression symptoms (*p* < 0.05, FDR corrected) have been reported previously in both clinical and non-clinical populations^26,27^, while associations between anxiety or depression and mobility features have generally been insignificant^28–30^. We are also identifying differences between incoming and outgoing calls with respect to symptomatology in SZ, which is consistent with the literature^19^, although our results suggest different directions of correlation. This could be due to the underlying complexity in completely capturing social behavior, as many methods of social communication (e.g. third-party chat applications and social media) are not observed by current methods. Figure 3c, d in our results suggests that passive data can pick up on social rhythm differences between clinical and non-clinical populations, and a recent systematic review has found similar flipping with respect to sleep duration and distance traveled, other markers of mobility and social rhythm^19^.

There are several limitations with the study. First, data quality and missingness could have a significant effect on the results, and could be a result of poor engagement, technical issues with the smartphone or study app, third-party apps for messaging leading to uncaptured sociability features, or smartphones being turned off or left home for long periods of time. Missingness in the passive data may reduce the chance of capturing behavior that matches the active data and low engagement in self-report reduces possible connections back to clinical symptoms. Second, the finding that there were more significant correlations in SZ than HC could be due to the fact that the smartphone app was originally designed for individuals with schizophrenia, who have more severe and variable symptoms, and may be more sensitive to picking up on behavior and symptomatology in SZ than HC. In addition, SZ and HC were not matched for age, race, and education, potentially introducing confounding factors especially with regards to the effect of age on circadian rhythm^31^. Despite the limitations, our study represents one of the largest digital phenotyping studies in schizophrenia in terms of sample size and duration^32^.

Today’s smartphones are capable of collecting, storing, and processing vast quantities of data reflecting real-time mobility and sociability behaviors—although utilizing that data to improve clinical outcomes has remained nascent. Using this data to assess social rhythms in schizophrenia offers a practical application that provides clinicians a potentially useful metric to assess recovery or relapse and patients a means to self-monitor and understand how their daily routines may impact their symptoms.

## Methods

### Data collection platforms

In this paper, we define two types of collected data as being either active or passive. Active data are directly entered by participants and includes survey responses and cognitive task results. Passive data are collected either entirely in the background or with limited user input, and includes GPS, accelerometer, screen on/off, and call/text logs. Two research applications were installed on participants’ smartphones as part of an Institutional Review Board-approved study (2017P-000359, “An Observational study of Digital Technology for Monitoring of Serious Mental Illness, 9/27/2019) to assist in active and passive data collection, respectively, mindLAMP and Beiwe, which have been previously reported on^33,34^.

### Participants and data collection protocol

Smartphone-owning adults (≥18 years old) were recruited from the greater Boston area through the Massachusetts Mental Health Center in Boston, MA and general public advertising. The recruitment goal of 80 participants (40 with schizophrenia and 40 healthy controls) was established from the conclusion of a successful pilot study with 17 participants and prior studies using smartphones for digital phenotyping in schizophrenia^35^. A total of 92 participants from August 22, 2018 to February 13, 2019 were enrolled after signing written informed consent and 88 completed both first and second in-person visits. Of those 88, 45 had a clinical diagnosis of schizophrenia or schizoaffective disorder confirmed by their medical record (SZ) and 43 were healthy controls (HC). All patients in the study were in active treatment. There were no exclusion criteria related to co-morbid diagnoses. All participants owned a smartphone and were given a smartwatch for the duration of the study to assist in data collection. Demographic information can be found in Table 1.

**Table 1:** Participant Demographics.

	HC (*n* = 43)	SZ (*n* = 45)	*p*
Age	30.49 (15.69)	37.45 (14.57)	0.044
Gender			1
Male	22 (51.2%)	23 (51.1%)	
Female	21 (48.8%)	22 (48.9%)	
Race			<0.001
American Indian or Alaskan Native	0 (0.0%)	4 (9.3%)	
Asian	30 (69.8%)	1 (2.3%)	
Black or African-American	4 (9.3%)	12 (27.9%)	
Multiracial or Other	2 (4.7%)	2 (4.7%)	
White Caucasian	7 (16.3%)	24 (55.8%)	
Education			<0.001
4-year college graduate or higher	36 (83.7%)	16 (35.6%)	
High school graduate/GED	3 (7.0%)	12 (26.7%)	
Some college	4 (9.3%)	14 (31.1%)	
Some high school	0 (0.0%)	3 (6.7%)	

88 smartphone-owning study participants from the greater Boston area participated in this three-month smartphone study.

### Data collection protocol

Data were collected over a three-month period with two in-person visits at the beginning and end of the study. At the first study visit, participants are explained the types of data collected, sign informed consent, and complete several paper and pencil tests. Participants then install both apps on their smartphones for active and passive data collection. No study compensation was provided for app use or engagement. After three months, participants return for a final visit where they repeat the assessments and receive an overview of their collected data.

**Fig. 4:**
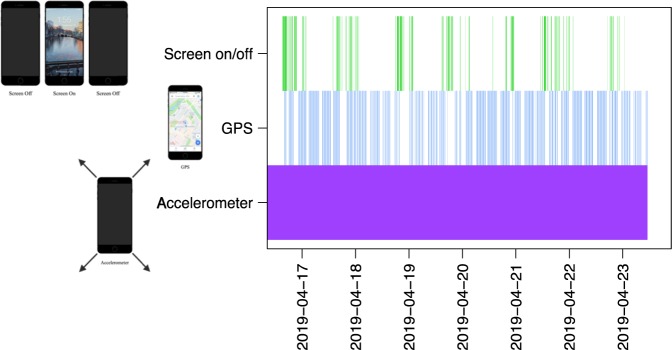
Sample of one-week passive data collection. This figure is a visualization of data collection frequency for three passive data streams throughout a week. Color indicates collection is on and white space indicates collection is off. In this example, accelerometer data are captured constantly, whereas GPS and screen on/off time (phone use) are captured less frequently.

During the three-month data collection period, participants were prompted on their smartphones to take 10 surveys per week: two each of mood (PHQ-9^36^), anxiety (GAD-7^37^), psychosis symptoms, sleep, and sociability. Throughout the study passive data were constantly collected via multiple passive data streams (GPS, accelerometer, screen on/off, and call/text logs) simultaneously (Fig. 4). Overall completion rates for smartphone surveys over the three months were 50% for SZ and 53% for HC. Missingness was assessed for GPS and accelerometer based on sampling rates and was found to be 72 and 60%, respectively.

### Data quality analysis

All data quality and subsequent analyses were performed using the R programming language (version 3.4, https://www.r-project.org/). Active data were analyzed to investigate survey completion rates and passive data were analyzed to calculate missingness based on a theoretical maximum number of data points generated from hardware sampling rates.

### Active vs passive data analysis

Active data were analyzed for mean and variance. Passive data were processed into interpretable features (e.g. time spent at home, number of incoming texts, etc.) for which individual-specific means and variances were computed. We used a statistical clustering method on both active and passive data variance as previously used in psychiatry research to categorize subjects based on large and/or complex data streams^25,38^. In this analysis, we used the standard method of *k*-means clustering to categorize SZ based on active data mean/variance and passive data mean/variance, then compared the clusters. The value for k was determined by using the Silhouette Method^39^ and the R packages “factoextra” and “Nbclust”^40^. In addition to clustering, we investigated associations between passive and active data features by creating a correlation matrix with every data stream. Correlations were conducted using the spearman method and corrected for multiple comparisons using FDR.

### Reporting summary

Further information on research design is available in the Nature Research Reporting Summary linked to this article.

## Supplementary information

nr-reporting summary

Supplementary Methods

## Data Availability

The data used in this study may be considered individually identifiable. Thus, it can only be shared with an ethics/IRB approved plan to ensure its deidentification and consent of the participants whom it was collected from.

## Code availability

Analysis code is available from the authors by request. Standard packages were used to generate results with no custom algorithms necessary for conclusions or replication.
