# Supplementary material for: Towards clinically actionable digital phenotyping targets in schizophrenia
Source: NPJ Schizophr. 2020 May 5;6:13. doi: 10.1038/s41537-020-0100-1 (PMC7200667; doi:10.1038/s41537-020-0100-1)
Supplement: Supplementary file 2 — Supplementary Methods [file 41537_2020_100_MOESM2_ESM.pdf]

Supplementary Methods

Hierarchical Clustering

To further interpret the correlation results between the SZ and HC and identify patterns in how certain features might be grouped together, we performed hierarchical clustering,[1] which begins with individual feature groups and iteratively aggregates similar features. Results of the cluster analysis are shown below. In both groups, sociability features generally clustered together whereas mobility features were distributed across all clusters. Active data features clustered more closely together in HC than SZ.

Feature Clusters in SZ. Blue = Passive Mobility, Green = Passive Sociability, and Red = Active

Cluster 1<br>Hometime, ProbPause, MinsMissing, CircdnRtn, WkEndDayRtn, text_responsiveness, sleep, medication	Cluster 2<br>DistTravelled, RoG, MaxDiam, MaxHomeDist, AvgFlightLen, StdFlightLen
Cluster 3<br>SigLocsVisited, SigLocEntropy, outgoing_texts, outgoing_textlengths, text_outdegree, incoming_texts incoming_textlengths, text_indegree, text_reciprocity, outgoing_calls, outgoing_calllengths, call_outdegree, incoming_calls, incoming_calllengths, call_indegree, call_reciprocity, call_responsiveness	Cluster 4<br>AvgFlightDur, StdFlightDur, social, psychosis, depression, anxiety

Feature Clusters in HC. Blue = Passive Mobility, Green = Passive Sociability, and Red = Active

Cluster 1<br>Hometime, ProbPause, MinsMissing, CircdnRtn, WkEndDayRtn, outgoing_texts, outgoing_textlengths, text_outdegree, incoming_texts, incoming_textlengths, text_indegree, text_reciprocity, outgoing_calls, outgoing_calllengths, call_outdegree, incoming_calls, incoming_calllengths, call_indegree, call_reciprocity, call_responsiveness	Cluster 2<br>DistTravelled, RoG, MaxDiam, SigLocsVisited, AvgFlightLen, StdFlightLen, SigLocEntropy, text_responsiveness, sleep, social, psychosis, depression, anxiety
Cluster 3<br>MaxHomeDist	Cluster 4<br>AvgFlightDur, StdFlightDur

References

1. Ward Jr, J. H. Hierarchical grouping to optimize an objective function. *J. Am. Stat. Assoc.* **58**, 236–244 (1963).
